# Dose and time response of ruminally infused algae on rumen fermentation characteristics, biohydrogenation and *Butyrivibrio* group bacteria in goats

**DOI:** 10.1186/s40104-016-0080-1

**Published:** 2016-04-07

**Authors:** Honglong Zhu, Veerle Fievez, Shengyong Mao, Wenbo He, Weiyun Zhu

**Affiliations:** Jiangsu Key laboratory of Gastrointestinal Nutrition and Animal Health, Laboratory of Gastrointestinal Microbiology, College of Animal Science and Technology, Nanjing Agricultural University, Nanjing, 210095 China; Department of Animal Production, Ghent University, Melle, 9090 Belgium

**Keywords:** Algae, Biohydrogenation, Goat, Hydrogenating bacteria

## Abstract

**Background:**

Micro-algae could inhibit the complete rumen BH of dietary 18-carbon unsaturated fatty acid (UFAs). This study aimed to examine dose and time responses of algae supplementation on rumen fermentation, biohydrogenation and *Butyrivibrio* group bacteria in goats.

**Methods:**

Six goats were used in a repeated 3 × 3 Latin square design, and offered a fixed diet. Algae were infused through rumen cannule with 0 (Control), 6.1 (L-Alg), or 18.3 g (H-Alg) per day. Rumen contents were sampled on d 0, 3, 7, 14 and 20.

**Results:**

H-Alg reduced total volatile fatty acid concentration and acetate molar proportion (*P* < 0.05), and increased propionate molar proportion (*P* < 0.05), whereas L-Alg had no effect on rumen fermentation. Changes in proportions of acetate and propionate in H-Alg were obvious from d 7 onwards and reached the largest differences with the control on d 14. Algae induced a dose-dependent decrease in 18:0 and increased *trans*-18:1 in the ruminal content (*P* < 0.05). H-Alg increased the concentrations of *t*9, *t*11-18:2 and *t*11, *c*15-18:2 (*P* < 0.05). L-Alg only seemed to induce a transient change in 18-carbon isomers, while H-Alg induced a rapid elevation, already obvious on d 3, concentrations of these fatty acid rose in some cases again on d 20. Algae had no effect on the abundances of *Butyrivibrio* spp. and *Butyrivibrio proteoclasticus* (*P* > 0.10), while H-Alg reduced the total bacteria abundance (*P* < 0.05). However, this was induced by a significant difference between control and H-Alg on d 14 (-4.43 %). Afterwards, both treatments did not differ as increased variation in the H-Alg repetitions, with in some cases a return of the bacterial abundance to the basal level (d 0).

**Conclusions:**

Changes in rumen fermentation and 18-carbon UFAs metabolism in response to algae were related to the supplementation level, but there was no evidence of shift in ruminal biohydrogenation pathways towards *t*10-18:1. L-Alg mainly induced a transient effect on rumen biohydrogenation of 18-carbon UFAs, while H-Alg showed an acute inhibition and these effects were not associated with the known hydrogenating bacteria.

**Electronic supplementary material:**

The online version of this article (doi:10.1186/s40104-016-0080-1) contains supplementary material, which is available to authorized users.

## Background

Ruminant-derived products are the major source of conjugated linoleic acid (CLA) in the human diet [1]. However, rumen biohydrogenation (BH) of dietary 18-carbon unsaturated fatty acids (UFAs) limits the availability of health-associated CLA in ruminant meat and milk. During BH process, a wide range of the 18-carbon unsaturated transient intermediates, such as *t*11-18:1, are formed with 18:0 being the end product [2, 3]. The ruminal formation of *t*11-18:1 is desirable because it can serves as a substrate for endogenous synthesis of *c*9, *t*11 CLA in the mammary gland and in muscle tissue [4, 5].

Previous studies in ruminants reported that supplementation of marine algae rich in 20:5n-3 [eicosapentanenoic acid (EPA)] and/or 22:6n-3 [docosahexaenoic acid (DHA)] could effectively inhibit the complete rumen BH of dietary 18-carbon UFAs [6, 7], result in rumen accumulation of *t*11-18:1, and finally elevate *c*9, *t*11 CLA in milk [8] and meat [9]. Incomplete BH of UFAs has been associated with the changes in the rumen bacterial community. Several recent studies reported that some as yet uncultured bacteria phylogenetically classified as *Prevotella*, *Anaerovoax*, *Lachnospiraceae* incertae sedis, unclassified *Ruminococcaceae*, and unclassified *Clostridiales* may involved in the BH of UFAs [7, 10–12]. However, the *Butyrivibrio* group is still thought to be the most active hydrogenating bacteria in the rumen [6, 10]. The *Butyrivibrio* group has been classified into two distinct groups: A and B [2]. The group A bacteria, an ill-defined taxon, including *B. fibrisolvens*, *B. hungatei*, and several *Pseudobutyrivibrio* spp. [6, 13], hydrogenate the 18:2 n-6 or 18:3 n-3 to 18:1. The group B bacteria, of which the phylogenetic position is very close to *B. proteoclasticus* [14–16], reduce the same fatty acids (FAs) to 18:0. Despite several studies investigating the microbiology of FAs metabolism in dairy cows [6] or dairy sheep [7], little information is available on BH in the rumen of goats [17]. Particularly, in vivo studies are lacking, although inter-species differences in ruminal lipid metabolism between the cow and small ruminants have been proposed [18].

Analysis of temporal changes in milk FAs composition has provided evidence that algae supplementation might result in time-dependent changes in ruminal BH [19, 20]. Indeed, Toral et al. observed this phenomenon by supplying fish oil plus sunflower oil in the diet of sheep [21], but the information on time response was limited to 10 d only. Therefore, in the present study, the effects of algae on temporal changes in rumen fermentation and BH intermediates in goats were examined based on analysis of samples collected after 0, 3, 7, 14, and 20 d. Moreover, the effects of algae on the *Butyrivibrio* group bacteria were also evaluated.

## Methods

### Animals, experimental design, and diets

All surgical and animal care procedures throughout the study followed protocols approved by Chinese Science and Technology Committee Experimental Animal Care and Use guidelines (1998). Six Boer crossbred wether goats (18.40 ± 0.95 kg body weight) fitted with ruminal cannula were used in a repeated 3 × 3 Latin square design with 21 d for each experimental period and 14 d interval between experimental periods. All animals were housed in individual pens with free access to fresh water. To avoid the potentially negative impact of the reduced feed intake on variation in passage rate from the rumen, animals were fed at a restricted level. The daily diet consisted of 366 g *Leymus chinensis* hay and 244 g of one of the three concentrates (C0, C1, and C3, respectively): 1) 244.0 g C0 plus 0.0 g algae (0 g/kg DM; Control); 2) 237.9 g C1 plus 6.1 g algae (10 g/kg DM; L-Alg); and 3) 225.7 g C3 plus 18.3 g algae (30 g/kg DM; H-Alg). Algae powder (*Schizochytrium* sp.; Xiamen Huison biotech Co., Ltd, Xiamen, China) used in this study contained a similar amount of DHA (19.0-20.0 % DM algae) as algae products used in other studies [6, 8]. Experimental treatments were designed to be within the range of algae doses evaluated previously in dairy cows [6] and sheep [7]. Algae suspension with 30 mL sterile distilled water was prepared in 50-mL syringes and infused into rumen via the rumen cannula (cannula diameter of 3 cm) before feeding.

Diets were formulated to be isonitrogenous according to the Feeding Standard of Meat Goats of the Ministry of Agriculture of the People’s Republic of China (NY/T 816-2004) [22] to meet the maintenance requirements for growing goats. To ensure complete daily consumption with a constant forage:concentrate ration of 60:40 [dry matter (DM) basis], feed offer were restricted with 90 % of the maintenance requirements. The diet (610 g DM per goat) was offered in equal amounts twice daily (0700 and 1700). Hay always was offered 15 min after concentrate feeding. Ingredients of concentrates and chemical analysis and fatty acid profile of concentrates, *Leymus chinensis* hay, and algae are given in Table 1.

**Table 1:** Composition of concentrates and proximate analysis^a^ and fatty acid profile of three concentrates, *Leymus chinensis* hay, and algae

	Concentrate (C)				
	C0	C1	C3	hay	Algae
Ingrediets, g/kg DM					
Corn grain	500	576	622		
Soybean meal	317	257	276		
Wheat bran	125	106	37.2		
Limestone	20.0	18.8	17.9		
CaHPO_4_ · 2H_2_O	10.0	13.8	17.7		
Salt	12.8	13.1	13.8		
Premix^b^	15.0	15.4	16.2		
Chemical composition, g/kg DM					
DM	887	870	878	916	980
NDF	115	109	89.0	569	-
CP	215	189	192	74.0	150
Starch	405	438	436	-	ND
EE	27	27	27	36	609
Fatty acid composition, g/kg FAME					
12:0	0.32	0.24	0.27	7.08	3.36
14:0	1.51	1.16	1.31	55.7	68.3
16:0	153	148	137	186	438
18:0	34.9	30.9	30.4	15.0	11.1
*trans*-18:1	7.23	6.36	4.79	5.87	0.47
*c*9-18:1	209	220	230	25.2	4.32
18:2 n-6	460	476	471	133	9.04
18:3 n-3	42.5	37.3	33.8	260	1.90
20:0	3.70	3.70	3.73	10.5	1.52
20:5 n-3	ND	ND	ND	ND	0.45
22:5 n-3	1.26	0.93	0.88	6.58	2.09
22:6 n-3	0.41	0.26	0.27	1.36	343

*ND* not detected; -, not measured

^a^Chemical composition of diets were calculated to contain (g/kg DM): DM (900, 901, 903), NDF (387, 384, 374), CP (120,120, 120), EE (32.0, 38.4, 49.7), for the Control, L-Alg and H-Alg diets, respectively.

^b^Premix contained on a DM basis (per kg): Vitamin A ≥ 1,100,000 IU, Vitamin D ≥ 300,000 IU, Vitamin E ≥ 3,200 IU, Fe ≥ 7 g, Cu ≥ 1.2 g, Mn ≥ 5 g, Zn ≥ 8 g, I ≥130 mg, Se ≥ 27 mg, Co ≥45 mg

### Chemical analyses

Samples of Hay, concentrates (C0, C1 and C3), and algae powder were analyzed for dry matter (DM), ether extract (EE), crude protein (CP) according to AOAC (1995) [23]. CP was calculated as Kjeldahl N*6.25. Starch content was determined following the enzymatic method described by Karkalas [24]. Neutral detergent fiber (NDF) was determined by Van Soest et al.’s method [25] in the presence of SDS and heat-stable α-amylase using Ankom Fiber Analyzer (Ankom Technology, Fairport, NY), and NDF was inclusive of residual ash.

### Sampling

Approximately 60 mL of rumen content were collected from multiple sites within the rumen of each animal at 0, 2, 4, 6, and 9 h after morning feeding on d 0, 3, 7, 14, and 20 during each period. The pH was measured immediately using a portable pH-meter (Ecoscan pH5, Singapore). After thorough mixing, a portion of rumen content (approximately 40 mL) was strained through four layers of cheesecloth. One mL of strained ruminal fluid was mixed with 0.2 mL of freshly prepared 25 % (w/v) metaphosphoric acids for volatile fatty acids (VFAs) analysis through a gas chromatograph (GC-14B, Shimadzu, Kyoto, Japan) equipped with a flame ionization detector and a capillary column: 30 m × 0.32 mm × 0.25 μm film thickness (Column No.34292-07B; Supelco, Bellefonte, PA, USA) [26]. To obtain overall daily measurements, rumen content of different sampling times on each day were pooled in equal volume according to the previous study [6]. After thorough mixing, the mixed sample was dispensed in tubes. An aliquot of approximately 6 mL of the pooled sample was used for microbial analysis. Another aliquot of approximately 20 mL was freeze-dried before FA analysis. All samples were stored at -20 °C until submitted for analysis.

### Rumen FA extraction, methylation and analysis

Total FA in freeze-dried rumen content (0.200 g) was exacted with chloroform/methanol (2:1, v/v) as described by Chow et al [27]. Tridecanoic acid (19:0, Nu-Chek Prep, Inc. USA) was added as the internal standard. The exacted FAs were methylated with 2 mL of 0.5 mol/L NaOCH_3_/MeOH at 60 °C for 30 min, followed by 2 mL of 14 % boron trifluoride in methanol at 60 °C for 30 min. The fatty acid methyl esters (FAME) were extracted with 3 mL and 2 mL hexane and evaporated to dryness under nitrogen at 60 °C. Residue was dissolved in 1 mL hexane and analyzed by gas chromatograph (Shimadzu GC-2010) on a CP-Sil88 capillary column (100 m × 0.25 mm × 0.2 μm) for FAME analysis. The FAME peaks were identified by comparison of their retention times with authentic reference standards (GLC-463, CLA U-60-M, CLA U-61-M, Nu-Chek- Prep Inc., USA). 1 μL of FAME was injected at a 1:3 split ratio. The injector and detector temperatures were maintained at 250 °C. The oven temperature was held at 120 °C for 8 min, increased at 10 °C/min to 190 °C (held for 15 min), then increased at 2 °C/min to a final temperature of 215 °C (held for 35 min). Ultra-pure nitrogen was used as carrier gas at constant inlet pressure (276.4 kPa). Satisfactory separation of most *cis*- and *trans*-18:1 isomers was obtained and these were identified by their order of elution in a single chromatographic run [28]. However, the *t*10-18:1 and *t*11-18:1 isomers were not well separated in some samples. In order to get the good separation of *t*10-18:1 and *t*11-18:1, a second chromatographic run was performed as described by Alves and Bessa [29]. The hydrogen was used as the carrier gas at 1 mL/min constant flow and the split ratio was 1:30.

### DNA extraction

*B. fibrisolvens* (DSM3071) from Rowett Research Institute UK was cultivated anaerobically under CO_2_ for 48 h at 39 °C in M8 medium without the addition of N-acetyl-D-glucosamine [30]. Genomic DNA was extracted from *B. fibrisolvens* (DSM3071) and ruminal content with phenol-chloroform as described by Zoetendal et al [31]. DNA was checked by electrophoresis on 1.2 % agarose gel containing GoldviewTM (SaiBaiSheng, Shanghai, China) and quantified using a Nano-drop spectrophotometer ND-1000 UV-Vis (Thermo Fisher Scientific, Inc., 212 USA).

### qPCR

All qPCR reactions were performed on an ABI Prism 7300 Sequence Detection System associated with Sequence Detection Software V1.2 (Applied Biosystems, USA). Each qPCR reaction was performed in triplicate. Average values were calculated for these replicates, and considered as one value for statistical analysis. The 16S rRNA gene-targeted primer sets used in this study: forward primer-5’-CGG TGA ATA CGT TCY CGG-3’, reverse-5’-GGW TAC CTT GTT ACG ACT T-3’ for total bacteria [32], forward-5’-GYG AAG AAG TAT TTC GGT AT-3’, reverse-5’--CCA ACA CCT AGT ATT CAT C-3’ for *Butyrivibrio* spp. [6], forward-5’-TCC GGT GGT ATG AGA TGG GC-3’, reverse-5’-GTC GCT GCA TCA GAG TTT CCT-3’- plus molecular beacon -5’-FAM-CCG CTT GGC CGT CCG ACC TCT CAG TCC GAG CGG-DABCYL-3’- for *B. proteoclasticus* [33]. The reaction procedures were performed as described by Lv et al [17]. Standard curves for total bacteria, *Butyrivibrio* spp., and *B. proteoclasticus* were respectively generated with serial diluted 16S rRNA gene amplicons obtained from the cultured *B. fibrisolvens* (DSM3071) and *B. proteoclasticus* gene clone A23 (GenBank: HQ326602). The amplicons were quantified using a Nano-drop spectrophotometer ND-1000 UV-Vis before dilution (Thermo Fisher Scientific, Inc., 212 USA).

Amplification efficiency of all quantitative PCR was calculated as follows: efficiency = [10^(-1/slope)^-1]. The real-time PCR efficiencies of total bacteria, *Butyrivibrio* spp. and *B. proteoclasticus* primers were 1.04 (slope = - 3.30; R^2^ = 0.99), 0.94 (slope = - 3.47; R^2^ = 0.99) and 0.97 (slope = - 3.30; R^2^ = 0.99), respectively.

### Statistical analysis

Before statistical analysis, average values were calculated for pH and VFAs at 0, 2, 4, 6, and 9 h per sampling day. All data for samples on d 0, d 3, d 7, d 14 and d 20 were analyzed by repeated measures analysis using the PROC MIXED of SAS (version 9.1, SAS Institute Inc., NC, USA) and assuming a first-order autoregressive covariance structure. The analyzed model Y_ikln_ = μ + T_i_ + D_k_ + P_l_ + T × D_ik_ + T × P_il_ + D × P_kl_ + (b + φ_k_) X_kl_ + G_n_ + e_ikln_,where μ was the overall mean, T_i_ the effect of algae treatment based on a the post-hoc test (Control = 1, L-Alg = 2, and H-Alg = 3), D_k_ the effect sampling day, P_l_ the effect of period, T × D_ik_, T × P_il_ and D × P_kl_ the interaction between formerly mentioned factors, b the common regression coefficient of initial value X_kl_, φ_k_ the slope deviation of the kth diet from common slope b, X_kl_ the initial value on d 0 (covariate), G_n_ the random effect of goats, e_ikln_ the residual error. Least squares means (adjusted for covariance) are reported throughout. Pearson correlation was analyzed using PROC CORR procedure to investigate the relationship between the log copies of qPCR of *B. proteoclasticus* and the concentration of 18:0 in the rumen content. Differences were declared significant at *P* < 0.05, and a value of *P* ≤ 0.10 was considered to reflect a trend towards significance.

## Results

### Rumen fermentation

A low level of algae supplementation (L-Alg) did not affect rumen fermentation (Table 2), whereas high level of algae (H-Alg) increased rumen pH (*P* = 0.01), reduced total VFAs (TVFAs) concentration (*P* = 0.03) with a shift towards propionate at the expense of acetate (*P* < 0.05).

**Table 2:** Effect of algae infusion on rumen fermentation characteristics

	Treatments^a^			*P*-value^b^			
Items	Control	L-Alg	H-Alg	SEM	A	T	A × T
pH	6.25^c^	6.26^c^	6.36^d^	0.021	0.01	0.36	0.42
TVFAs, mmol/L	88.85^d^	87.71^d^	81.46^c^	0.978	0.03	0.70	0.75
Molar proportion, %							
Acetate	73.60^d^	73.90^d^	70.50^c^	0.350	<0.01	0.07	0.02
Propionate	14.21^c^	14.22^c^	16.17^d^	0.198	<0.01	0.02	0.01
Butyrate	8.70^c^	8.34^c^	9.56^d^	0.158	0.05	0.06	0.10
Isobutyrate	1.48	1.49	1.49	0.051	0.68	0.06	0.10
Isovalerate	1.88	1.92	2.00	0.078	0.72	0.14	0.03
Valerate	0.58	0.61	0.67	0.016	0.16	0.32	0.51

*TVFAs;* total volatile fatty acids

^a^Treatments: rumen infusion of 0.0 g/d algae (Control); rumen infusion of 6.1 g/d algae (L-Alg); rumen infusion of 18.3 g/d algae (H-Alg)

^b^Probability of linear effect of algae infusion (A), time effect following algae infusion (T), and their interaction (A × T)

^c, d^Means within a row with different superscripts differ significantly at *P* < 0.05

**Fig. 1:**
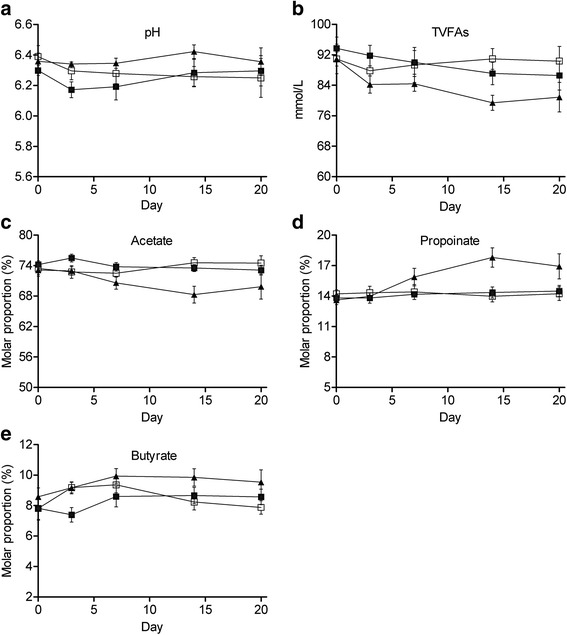
Changes in pH (**a**), TVFAs (**b**) (mmol/L) and molar proportions (%) of acetate (**c**), propionate (**d**) and butyrate (**e**) in goats fed diets with rumen infusion 0.0 g/d algae (Control, □), 6.1 g/d algae (L-Alg, ■), and 18.3 g/d algae (H-Alg, ▲)

All the rumen fermentation parameters for goats in control and L-Alg remained relatively constant throughout the experiment (Fig. 1). For goats with H-Alg, however, algae caused a significant effect on TVFAs, with an immediate decrease in the first 3 days, which continued to drop until d 14, the maximum difference between H-Alg and control observations was 12.6 %. Molar proportions (%) of acetate decreased (*P* = 0.07) in H-Alg, while propionate (*P* = 0.02) and butyrate (*P* = 0.06) progressively increased, reaching a minimum or maximum.

### Rumen FA composition

**Fig. 2:**
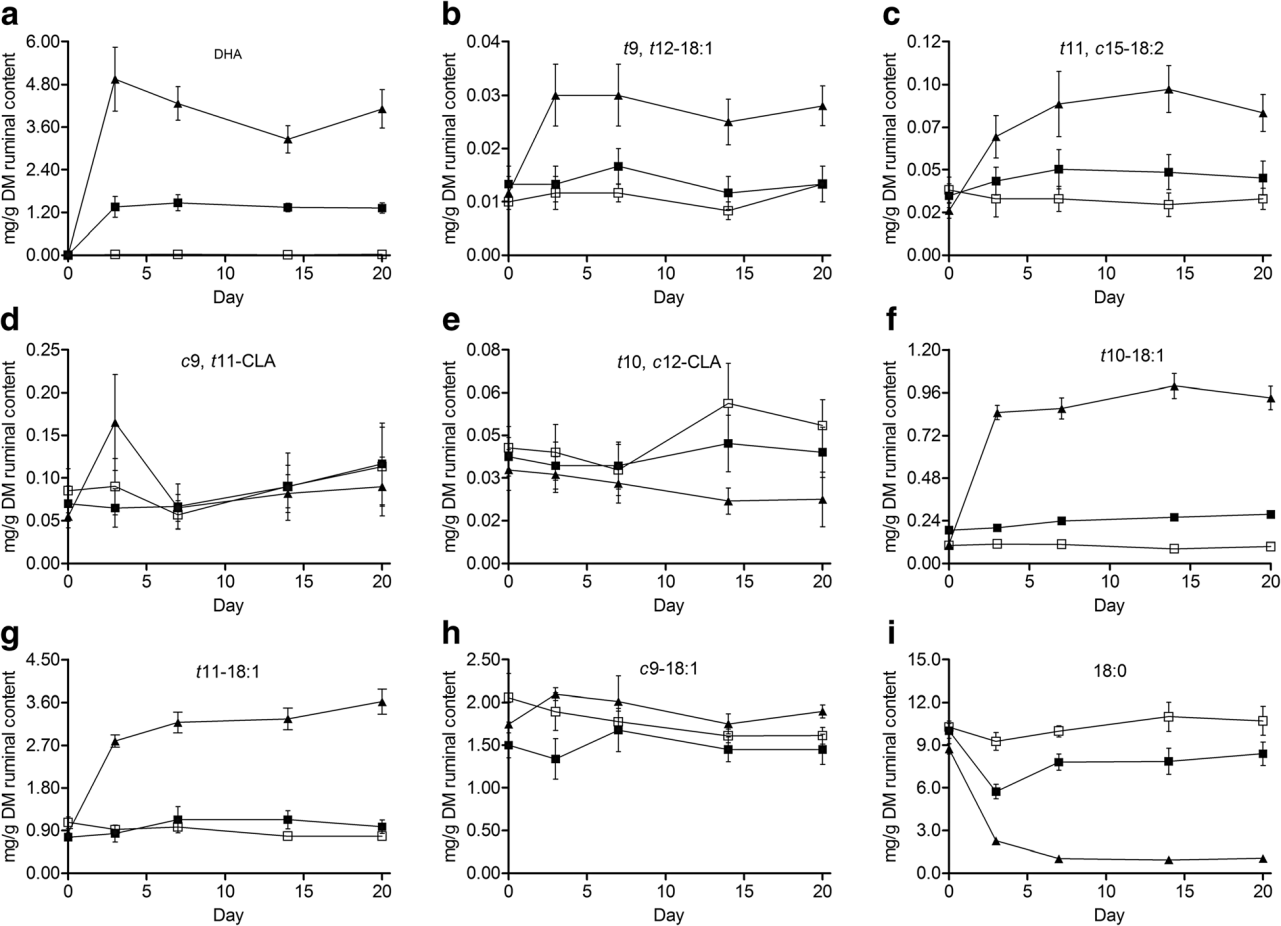
Changes in the concentrations (mg/g DM ruminal content) of 22:6 n-3 (**a**), *t*9, *t*12-18:2 (**b**), *t*11, *c*15-18:2 (**c**), *c*9, *t*11-CLA (**d**), *t*10, *c*12-CLA (**e**), *t*10-18:1 (**f**), *t*11-18:1 (**g**), *c*9-18:1 (**h**) and 18:0 (**i**) in goats fed diets with rumen infusion 0.0 g/d algae (Control, □), 6.1 g/d algae (L-Alg, ■), and 18.3 g/d algae (H-Alg, ▲)

Algae largely changed ruminal FAs composition (Table 3), with most FAs showing time responses to algae infusion (Fig. 2 and Additional file 1: Figure S1), although L-Alg and H-Alg most often differed. As expected, hardly any changes occurred in the control throughout the experiment period.

**Table 3:** Effect of algae infusion on fatty acid profile of the ruminal content

	Treatments^a^			*P*-value^b^			
Fatty acid, mg/g DM	Control	L-Alg	H-Alg	SEM	A	T	A × T
12:0	0.10	0.09	0.10	0.002	0.99	0.79	0.12
13:0	0.07^g^	0.09^h^	0.07^g^	0.002	<0.01	0.53	0.14
14:0	0.67^g^	0.82^h^	1.12^i^	0.033	<0.01	0.43	0.14
15:0	1.20	1.17	1.18	0.044	0.83	<0.01	0.13
16:0	5.17^h^	7.43^g^	13.21^i^	0.366	<0.01	0.35	0.15
17:0	0.20^i^	0.17^h^	0.15^g^	0.003	<0.01	0.03	0.21
18:0	10.03^i^	7.34^h^	1.66^g^	0.413	<0.01	0.09	<0.01
*t*5 -18:1	<0.01^g^	0.01^h^	0.02^i^	0.001	<0.01	<0.01	<0.01
*t*6+ *t*7+ *t*8-18:1	0.05^g^	0.08^h^	0.10^i^	0.003	<0.01	0.66	0.01
*t*9-18:1	0.03^g^	0.14^h^	0.46^i^	0.020	<0.01	0.42	0.19
*t*10-18:1	0.21^g^	0.22^g^	1.01^h^	0.036	<0.01	0.02	0.04
*t*11-18:1	0.71^g^	1.13^h^	3.26^i^	0.114	<0.01	0.10	0.11
*t*12-18:1	0.03^g^	0.12^h^	0.41^i^	0.018	<0.01	0.38	0.02
*t*13 + *t*14-18:1	0.02^g^	0.29^h^	0.81^i^	0.035	<0.01	0.15	0.02
*t*16 + *c*14-18:1	0.04^g^	0.16^h^	0.21^i^	0.010	<0.01	<0.01	<0.01
*c*9-18:1	1.58^g^	1.60^g^	1.95^h^	0.051	0.03	0.15	0.44
*c*11-18:1	0.12^g^	0.12^g^	0.23^h^	0.006	<0.01	0.002	0.26
*c*12-18:1	0.02^g^	0.03^h^	0.05^i^	0.002	<0.01	0.81	0.17
*c*13-18:1	<0.01^g^	0.01^h^	0.03^i^	0.001	<0.01	0.05	0.01
*t*9, *t*12-18:2	0.01^g^	0.01^g^	0.04^h^	0.001	<0.01	0.24	0.72
*c*9, *t*12-18:2	0.03	0.02	0.03	0.001	0.59	0.03	0.72
t9, c12-18:2	0.04	0.08	0.07	0.002	0.72	<0.01	0.10
*t*11, *c*15-18:2	0.03^g^	0.03^g^	0.09^h^	0.003	<0.01	0.08	0.05
18:2 n-6	1.96	1.59	1.35	0.085	0.09	0.60	0.91
18:3 n-3	0.35	0.30	0.26	0.020	0.09	<0.01	0.14
*c*9, *t*11-CLA	0.07	0.09	0.12	0.008	0.68	<0.01	0.01
*t*10, *c*12-CLA	0.04^h^	0.04^h^	0.03^g^	0.002	0.03	0.17	0.13
22:6 n-3	0.08^g^	1.36^h^	4.12^i^	0.199	<0.01	0.10	0.08
*Trans*-18:1^c^	1.03^g^	2.15^h^	6.19^i^	0.229	<0.01	0.52	0.18
*Cis*-18:1^d^	1.75^g^	1.80^h^	2.29^i^	0.056	<0.01	0.14	0.43
Non-conjugated 18:2^e^	0.09^g^	0.14^h^	0.19^i^	0.007	0.01	0.08	0.74
Conjugated 18:2^f^	0.11	0.12	0.15	0.014	0.91	<0.01	0.02

^a^Treatments: rumen infusion of 0.0 g/d algae (Control); rumen infusion of 6.1 g/d algae (L-Alg); rumen infusion of 18.3 g/d algae (H-Alg)

^b^Probability of linear effect of algae infusion (A), time effect following algae infusion (T), and their interaction (A × T)

^c^ *Trans*-18:1, ∑ [*t*5-18 + (*t*6 + *t*7 + *t*8)-18:1 + *t*9-18:1 + *t*10-18:1 + *t*11-18:1 + *t*12-18:1 + (*t*13 + *t*14)-18:1 + (*t*16 + *c*14-18:1)]

^d^ *Cis*-18:1, ∑ (*c*9-18:1 + *c*11-18:1 + *c*12-18:1 + *c*13-18:1)

^e^Nonconjugated 18:2, ∑ (*t*9, *t*12-18:2 + *c*9, *t*12-18:2 + t9, *c*12-18:2 + *t*11, *c*15-18:2)

^f^Conjugated 18:2, ∑ (*c*9, *t*11-CLA + *t*10, *c*12-CLA)

^g, h, i^ Values within a row with different superscripts differ significantly at *P* < 0.05

Algae linearly increased the concentration of DHA in ruminal content (*P* < 0.01), which showed sharp increases on d 3 after algae infusion, and then remained constantly at a greater level as compared with the control (Fig. 2a). At the end of the experiment, the concentrations of DHA were 42- and 134-fold greater in ruminal content of goats with L-Alg and H-Alg than goats fed the control diet.

Algae had no effect on the concentration of total conjugated 18:2 in the rumen (*P* = 0.91), while total non-conjugated 18:2 linearly increased, mainly due to the substantial and immediate increase of *t*9, *t*12-18:2 and *t*11, *c*15-18:2 (*P* < 0.05). The concentration of *t*9, *t*12-18:2 only temporarily (d 7) was greater than the control in L-Alg. For goats receiving H-Alg, however, *t*9, *t*12-18:2 sharply increased on d 3, after which this remained relatively constant (Fig. 2b). The immediate response to algae in *t*11, *c*15-18:2 was maintained after the first week of supplementation for L-Alg and slightly increased further until d 14 when supplementing H-Alg (Fig. 2c). The concentration of *c*9, *t*11-CLA appeared to be relatively stable in the control and L-Alg, but temporarily increased in H-Alg, with a transient peak on d 3 (Fig. 2d). L-Alg did not influence the concentration of *t*10,*c*12-CLA, while data from the H-Alg showed a significant decrease (*P* < 0.05), which was mainly due to a sudden increase from d 14 onwards in the control as H-Alg did not induce changes with time (Fig. 2e).

Algae linearly increased the concentrations of all *trans*-18:1 isomers, particularly *t*11-18:1, with the H-Alg provoking a much more pronounced effect (*P* < 0.01; Table 4). The concentration of *t*11-18:1 increased in H-Alg with the steepest increase occurring between d 0 and d 3, although concentrations further raised till d 20, when levels were 365 % greater as compared with the control (Fig. 2g). The concentration of *t*10-18:1 in H-Alg showed an accumulation, but no shift in rumen BH toward *t*10-18:1 at the expense of *t*11-18:1 was observed (Table 4). Other *trans*-18:1 isomers in H-Alg also showed a steep increase during the first 3 d, with a minor further increase on d 20 (expect for *t*5-18:1 and *t*13 + *t*14-18:1; Additional file 1: Fig. S1). In contrast, L-Alg responses seemed transient with a maximum concentration on d 7, after which concentrations dropped between d 7 and d 20. Differences in the concentration of *c*9-18:1 among the groups were observed at the start of experiment (d 0), while no difference was shown at any time among all groups after 1 week algae infusion (Fig. 2h). *c*11-18:1 was not affected by L-Alg, while H-Alg significantly increased *c*11-18:1 (Table 4), showing a rapid increase on d 3, remaining at a stable level between d 7 and d 14, but further raised on d 20. The concentrations of *c*12-18:1 and *c*13-18:1 followed the same pattern of temporal change as mentioned above for *trans*-18:1 isomers.

**Table 4:** Effect on algae infusion on the rumen bacterial abundance

	Treatments^a^			*P*-value^b^			
Items	Control	L-Alg	H-Alg	SEM	A	T	A × T
Total bacteria^c^	9.47^e^	9.45^e^	9.17^d^	0.038	<0.01	0.07	0.42
*Butyrivibrio* spp.^c^	6.34	6.36	6.37	0.026	0.99	0.02	0.15
*Butyrivibrio proteoclasticus* ^c^	4.87	4.64	4.54	0.053	0.24	0.04	0.67

^a^Treatments: rumen infusion of 0.0 g/d algae (Control); rumen infusion of 6.1 g/d algae (L-Alg); rumen infusion of 18.3 g/d algae (H-Alg)

^b^Probability of linear effect of algae infusion (A), time effect following algae infusion (T), and their interaction (A × T)

^c^Values are expressed in log_10_ number of 16S rRNA gene copies per mL rumen contents

^d, e^Values within a row with different superscripts differ significantly at *P* < 0.05

Algae linearly decreased the concentration of 18:0 (*P* < 0.01). A sharp decrease in 18:0 in response to algae was detected from the first measurement (d 3), but some recovery from d 7 onwards was observed for L-Alg (Fig. 2i). However, H-Alg resulted in an almost complete absence of 18:0 from d 7 onwards.

### Bacterial abundance

**Fig. 3:**
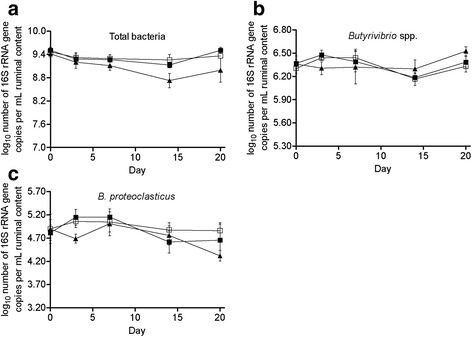
Changes in the abundance of total bacteria (**a**), *Butyrivibrio* spp. (**b**) and *B. proteoclasticus* (**c**) in goats fed diets with rumen infusion 0.0 g/d algae (Control, □), 6.1 g/d algae (L-Alg, ■), and 18.3 g/d algae (H-Alg, ▲)

L-Alg had no effect on the abundance of total bacteria (log_10_ number of 16S rRNA gene copies per mL ruminal content), while H-Alg reduced total bacterial abundance (*P* < 0.01; Table 4), although biologically relevant differences only occurred on d 14 (-4.43 %; Fig. 3a). Afterwards the control and H-Alg treatment did not differ due to an increased variation among H-Alg replicates, with bacterial abundance in the rumen of some goats returning on d 20 to the basal level of the start of the experiment (d 0). Algae infusion did not influence the abundance of *Butyrivibrio* spp. (*P* = 0.99) and *B. proteoclasticus* (*P* = 0.24).

## Discussion

### Rumen fermentation

Previous studies have demonstrated that algae can affect rumen fermentation in vitro in whethers [34]. In our study, algae infusion at 6.1 g/d (10 g/kg DM, L-Alg) had no effect on rumen pH and TVFAs concentration, whereas algae infusion at 18.3 g/d (30 g/kg DM, H-Alg) increased rumen pH, and decreased TVFAs concentration, which is in line with the general negative correlation between pH and TVFAs levels [35]. H-Alg induced an evident shift of the VFAs pattern, whereas no effect was observed with the L-Alg treatment. The different responses may relate to the amounts of algae supplemented and hence difference in the supply of long-chain (20+) polyunsaturated fatty acids (PUFAs). Also, others [21, 36] did not observe shifts when dairy sheep were fed a diet containing similar amounts of EPA + DHA as supplied through the L-Alg diet (approximately 2.0 g of DHA/kg DM). In contrast, in an earlier report [8], supplementation of algae in the concentrate at a rate of 9.35 g/kg DM of the same algae species (*Schizochytrium*) modified rumen fermentation pattern in dairy cows. This might be related to difference between ruminant species with small ruminants eventually having a greater tolerance for dietary PUFAs and/or the mode of algae supplementation (ruminal infusion vs incorporation in the concentrate). Indeed, data from our group [37] also indicated that similar amounts of EPA or/and DHA as supplemented through the H-Alg treatment (6.0 g of DHA/kg DM) altered rumen fermentation pattern in goats fed similar basal diets. It is further noticed that the response in acetate and propionate proportion did not seem acute, as indicated in Fig. 1, with changes only being apparent from d 7 onwards and shifts continuing until d 14.

### Rumen lipid metabolism

In the rumen, BH of 18:3 n-3 starts with an isomerization to yield *c*9, *t*11, *c*15-18:3, which is then sequentially hydrogenating to *t*11, *c*15-18:2, *t*11-18:1 and 18:0. Similarly, BH of 18:2 n-6 yield *c*9, *t*11-CLA, which then reduced to *t*11-18:1 and finally to 18:0. In recent years, algae, as a substitute for fish oil, proved to possess high effectiveness in the inhibition of rumen BH of 18-carbon UFAs [6, 7]. As expected, in our study, algae increased the concentration *t*11, *c*15-18:2 in the rumen, consistent with previous studies in dairy cows [6, 38] or sheep [7]. Results of the in vitro study by Vlaeminck et al. [39] found that *t*11, *c*15-18:2 accumulation seemed relatively independent: *t*11, *c*15-18:2 only accumulated in the presence of DHA, when removal of DHA, the accumulation of *t*11, *c*15-18:2 removed. Our results showed that *t*11,*c*15-18:2 was maintained after the first week of supplementation for L-Alg and slightly increased further until d 14 when supplementing H-Alg, suggesting that algae may be not lethal to *t*11, *c*15-18:2 producing bacteria in contrast to lethal bacteria converting 18:1 to 18:0. The *c*9, *t*11-CLA was the major 18:2 isomer in the rumen, which confirmed earlier reports, irrespective of dietary composition and ruminant species [7, 40]. However, earlier studies showed the inconsistent effects of algae supplementation on the concentration of *c*9, *t*11-CLA, with no effect [38], or increase [6], even decrease [41] in the rumen. In our study, a transient peak of *c*9, *t*11-CLA was observed on d 3 when supplementing the high algae dose (H-Alg), although algae had no effect on the concentration of *c*9, *t*11-CLA, suggesting that the growth and/or activity of *t*11-18:1 producing bacteria may be inhibited by algae supplementation at the start of experiment, and afterwards showed an adaptation to the algae supplementation. Several studies indicated that the syndrome of milk fat depression (MFD) in dairy cows or sheep fed high-concentrate diet was generally associated with the increase of *t*10, *c*12-CLA in the rumen of dairy cows or sheep. In the present study, the concentrations of *t*10, *c*12-CLA in the ruminal content were very low, with the average value at 0.04 mg/g. Furthermore, there was no clear effect on *t*10, *c*12-CLA (Fig. 2e), confirming the view that the mechanism for MFD induced by algae differs from MFD associated with concentrate-rich diets, and might be associated with increased production of other BH intermediates.

Previous studies in dairy cows have demonstrated that algae supplementation (9.35, 22, or 43 g/kg DM) results in a shift in ruminal BH toward increased formation of *t*10-18:1 at the expense of *t*11-18:1 [6, 41]. Measurements of milk fat composition in cows also showed that algae supplementation resulted in a rapid enhancement of *t*11-18:1 in milk fat that declined over time, which was associated with concomitant increases in *t*10-18:1 [8]. Generally, this shift in ruminal BH pathways might be associated with MFD, since *t*10-18:1 could inhibit mammary lipogenesis in the bovine [42]. In the present study, the both *t*10-18:1 and *t*11-18:1 isomers in the rumen content increased by algae infusion, but no significant shift from *t*11-18:1 to *t*10-18:1 was observed between the control and algae treatments. This result is consistency with the recent report of Toral et al. [7] on lactating sheep, who reported that supplementing the diet with 33 g/kg of DM of a mixture of sunflower oil (25 g/kg DM) and algae (8 g/kg DM) did not induce a shift in the ratio of *t*10-18:1 to *t*11-18:1 in rumen fluid, but the “*t*10-18:1 shift” was observed when applying higher amounts of algae (16 and 24 g/kg DM). These collective findings may suggest that the propensity for ‘*t*10-18:1 shift’ was lower in sheep or goat compared with cows [42], which also suggests inter-species difference.

One of the striking results of this study is related to the transient increase until d 7 of 18:1 isomers when applying the L-Alg treatment. Earlier a study [6] with similar dietary algae doses, supplied to dairy cows showed a rapid increase until day 6 which remained at a constant level afterwards. This further may indicate the difference in tolerance for dietary PUFAs between ruminant species, which was in accordance with Shingfield et al. [9] who suggested that rumen BH pathways in the small ruminants were less altered by dietary changes as compared with the cows. Despite the transient effect on 18:1 isomers with the L-Alg treatment, the concentration of 18:0 was lower than the control throughout the experimental period. Hence, the decrease in 18:0 primarily was related to accumulation of non-conjugated 18:2 isomers, such as *t*11, *c*15-18:2.

### Rumen bacterial abundance

L-Alg did not influence the abundance of total bacteria, which was in agreement with results by Boeckaert et al. [6] who found that dietary supplementation of algae in the concentrate (9.35 g/kg DM) didn’t affect on the abundance of total bacteria monitored on d 6, 13 and 20 after the start of the supplementation as compared with 2 d before the supplementation start in dairy cows. However, H-Alg did cause a decrease in total bacterial abundance. This indicates the importance of the algae supplementation level and is in accordance with our former suggestion on the importance of the dose to provoke a bacteriacidal effect. Furthermore, the overall difference in the abundance of total bacteria between the control and H-Alg mainly was provoked by a decrease on d 14, because abundance on d 20 returned to the basal level (d 0) for some cases. This suggests the bacteriadal effect to be provoked mainly by a continuous supplementation during a longer period (14 d) rather than to an acute effect, but that the rumen microbiota also may adapt to algae when supplemented over a longer period. Data from Belenguer et al. [43], using 30 g/kg DM of a mixture (1:2 wt/wt) of fish oil and sunflower oil supplementation, rather suggested an acute effect as the bacterial abundance decreased on d 3 compared with d 0 (control) in sheep, but the numbers actually recovered on d 10. The differences in time response may associate with the different source of the used PUFA in the current experiment compared with the experiment of Belenguer et al [43]. Oil rich in PUFAs may be accessible to rumen microbes as compared with algae powder eventually resulting in a more acute bacteriacidal effect.

Previous studies indicated that the *Butyrivibrio* group bacteria play a dominate role in the conversion of dietary 18:2 n-6 and 18:3 n-3 to 18:1. In our study, algae supplementation did not influence the abundance of *Butyrivibrio* spp., which in agreement with results by Boeckaert et al. who reported that *Butyrivibrio* spp. did not change in dairy cows supplemented with the same algae as used in the current study. A probable explanation is that this group of bacteria showed a higher tolerance to the toxicity of PUFAs as compared with other bacteria in the rumen [44]. Early studies indicate that *B. proteoclasticus* bacteria are able to efficiently convert 18:1 to 18:0 in vitro [15, 45]. In the present study, algae resulted in a linear decrease in the concentration of 18:0 and a linear accumulation in 18:1 with the increased levels of algae fusion, indicating that the conversion of 18:1 to 18:0 was inhibited by algae. However, our results show that there was a week correlation between the numbers of *B. proteoclasticus* and 18:0 (*R*^*2*^ = 0.14, *P* = 0.18), indicating *B. proteoclasticus* have a limited contribution in vivo to 18:0 formation in goats. Similarly, recent in vivo studies had shown a lower relevance of these species in rumen BH than initially thought [10, 11]. Also, we found that the relative abundances of *B. proteoclasticus* in total bacteria were very low in goats, with the average value at 0.002 %. Previous studies, however, reported that the relative abundances of the *B. proteoclasticus* in total bacteria were higher in dairy cows (7-9 %) [33] or sheep (0.18 %) [43]. Thus, the inter-species differences in the abundances of *B. proteoclasticus* exist clearly between ruminants. Nevertheless, a direct comparison among ruminants should be considered, and may provide an opportunity for better understanding the role for different microbial species involved in BH in the future.

### Intake and ingredients of concentrates

Previous studies have demonstrated that supplementing diets with algae often decreases DMI in sheep [46] or cows [8]. In the present study, goats consumed all the feeds offered throughout the experiment, which was probably related to animals being fed at a restricted level rather than an indication of algae supplements. Consistent with previous studies [17, 21], in order to ensure isonitrogenous nutrient among diets, the slightly difference in ingredients such as the amount of corn grain (starch content; 405, 438, 436 g/kg DM) also exist in the present study due to marine lipids supplementation. This difference may also affect the rumen metabolism and thus may hinder the attribution of some of the observed effect to the algae infusion.

## Conclusion

Algae infusion at 6.1 g/d (10 g/kg DM; L-Alg) via ruminal cannula did not influence rumen fermentation in goats, but increasing the amount to 18.3 g/d (30 g/kg DM; H-Alg) altered rumen fermentation towards propionate at the expense of acetate although this effect only was apparent after 1 week of infusion. Metabolism of 18-carbon UFAs in response to algae supplementation was also related to the level of its supplementation: L-Alg mostly induced a transient effect in rumen BH of 18-carbon UFAs, while an acute and persisting inhibition was provoked by H-Alg. However, there was no evidence of shift in ruminal biohydrogenation pathways towards *t*10-18:1. Algae did not affect the abundances of known hydrogenating bacteria. Results from the current study also might suggest that goats were more tolerant to dietary PUFAs than dairy cows but a direct comparison between species is required.

## Additional file

Additional file 1: Figure S1. Changes in the concentrations (mg/g DM ruminal content) of *t*5-18:1 (a), *t*6 + *t*7 + *t*8-18:1 (b), *t*9-18:1 (c), *t*12-18:1 (d), *t*13 + *t*14-18:1 (e), *c*11-18:1 (f), *c*12-18:1 (g), *c*13-18:1 (h) and *c*14 + *t*16-18:1 (i) in goats fed diets with rumen infusion 0.0 g/d algae (Control, □), 6.1 g/d algae (L-Alg, ■), and 18.3 g/d algae (H-Alg, ▲). (TIF 951 kb)
